# Social Representations of Formal Volunteers and Spontaneous Volunteers in Socio-Natural Disaster Risk Management Contexts

**DOI:** 10.3390/bs15040497

**Published:** 2025-04-09

**Authors:** Matías Peña-Garay, José Sandoval-Díaz, David Cuadra-Martínez

**Affiliations:** 1 Programa de Doctorado en Ciencias Sociales, Facultad de Ciencias Sociales y Económicas, Universidad Catolica del Maule, Talca 3460000, Chile; 2 Departamento de Ciencias Sociales, Facultad de Educación y Humanidades, Universidad del Bío-Bío, Chillán 3780000, Chile; jsandoval@ubiobio.cl; 3 Centro de Estudios Ñuble, Universidad del Bío-Bío, Chillán 3780000, Chile; 4 Departamento de Psicología, Universidad de Atacama, Copiapó 1530000, Chile; david.cuadra@uda.cl

**Keywords:** prosocial, socio-natural disaster risk management, altruistic behavior, social representations, volunteer

## Abstract

Background: Citizenship plays a fundamental role in the management of socio-natural disaster risk, especially given the increasing impact and frequency of these events. In this context, disaster response is marked by both formal and spontaneous volunteerism. Method: Using a non-probabilistic sample of 101 volunteers and comparing the social representation of formal volunteers with spontaneous volunteers, prototypical and categorical analyses of social representations were conducted. Results: Differences were identified between formal volunteers, whose social representation reflects a strong value-oriented and collaborative vision focused on social capital, and spontaneous volunteers, whose social representation is directed toward prosocial values, emotions, and the heroic actions associated with volunteerism. Conclusions: New avenues are proposed for exercising and strengthening formal volunteerism, accompanied by processes that enable the identification of common value axes and suitability for risk-related work. Additionally, the motivations and actions of spontaneous volunteers are discussed. Finally, sustained coordination is proposed among institutions involved in risk management, formal volunteers, and spontaneous volunteers to optimize human resource management in emergencies.

## 1. Introduction

Environmental changes and socio-natural disasters are strongly driven by the idea of modernity and, therefore, by the defense of the idea of progress and development, based on the arbitrary manipulation of natural resources. This perspective places the human species at the center of the phenomenon of climate change, suggesting that we are currently in the Anthropocene geological era, an era marked by human manipulation and control over the Earth (IPCC, 2018). Due to rising global average temperatures, constant and indiscriminate intervention in local biodiversity, extensive emissions distribution, and deficient resource and waste management, climate change has had a significant impact on the development of socio-natural disasters (Trischler, 2017).

According to the United Nations Office for Disaster Risk Reduction (UNDRR, 2015), there has been an 80 percent increase in the frequency and occurrence of these phenomena, with Latin America reporting an average of 40 major disasters annually over the last decade, placing the region second only to Asia in terms of frequency. In Chile, a significant impact has been observed, with the increased frequency and magnitude of various socio-natural disasters throughout the country. Notably, 50% of the Chilean population is exposed to three or more types of socio-natural disasters (Dilley, 2005). Among these events, forest fires and floods stand out, which have been exacerbated by increased climatic events such as extreme rainfall, drought, and severe heatwaves, making the region highly vulnerable to these incidents (Sandoval-Díaz et al., 2023).

In Latin American disaster response frameworks, first responders to emergencies are often citizens, with many of them being volunteers. As a result, volunteerism emerges as a key factor in disaster response, particularly in a country like Chile, where disaster volunteer responders are formalized, institutionalized, characterized, and widely recognized, carrying a strong sense of adherence, belonging, and camaraderie, as seen with Chilean firefighters (Cerna & Quilodrán, 2017). Additionally, from citizens not affiliated with formal volunteerism, cooperation behaviors and emergent practices arise, leading to spontaneous civic responses to socio-natural disasters in various areas of action, such as rescue, logistics, reconstruction, and more (Cárdenas-Briones, 2020).

Collaboration and coordination between spontaneous volunteer communities and formal volunteer institutions is crucial for facing and managing socio-natural disaster risk (Eriksson & Danielsson, 2022). However, it is observed that volunteer experience in disaster contexts can be heavily influenced by social, cultural, and emotional aspects, which may present challenges for risk management due to the difficulties in coordinating and managing formal and spontaneous volunteer forces (Fong-Lee & Vega-Saenz, 2021). This is due to the lack of connection and training for their actions in socio-natural disaster risk contexts, which can characterize the volunteer response as both a blessing and a challenge (Hartmann et al., 2020).

Disaster risk management is defined as a systematic approach and practice aimed at managing the uncertainty of damage that socio-natural disasters can cause, implementing policies and strategies for prevention, preparedness, response, and recovery in disaster scenarios (Hardy Casado et al., 2021). This process strengthens coping mechanisms, response capacities, and reduces damage. It is characterized by being comprehensive, strategic, and participatory, involving multiple stakeholders and programs (Al-Mueed et al., 2021). This concept helps decipher the complexities of human responses in disaster risk situations and provides a knowledge framework that not only reveals the dynamics of volunteer behavior in disaster contexts but also offers perspectives for optimally enhancing socio-natural disaster risk management, community resilience, and adaptation (Rosales-Veítia, 2021; Sandoval-Díaz et al., 2023).

Research in socio-natural disaster risk management has focused efforts on strengthening institutional frameworks based on policies and empowering affected communities, promoting resilience and preparedness approaches, while neglecting research on first responders, particularly volunteer responders, which is considered underrepresented in the literature (Flores & Najberg, 2021). A quantitative study on disaster management perception conducted by Yiwo et al. (2022) demonstrates that a statistically significant proportion of participants recognize the importance of citizen participation in the various phases of flood risk management. From this perspective, both citizens and volunteers are considered fundamental pillars in reducing the risk of socio-natural disasters. In this context, it becomes essential to deepen the understanding of volunteerism and the motivations that drive it in disaster scenarios. This entails analyzing how volunteer groups perceive their actions and exploring the psychosocial dimensions that characterize them. Such an approach would not only enable more efficient management of volunteer efforts but also allow for a clearer distinction between the dynamics of formal and spontaneous volunteer activities in the face of risk (Cuadra-Martínez et al., 2023).

Prosocial behavior has emerged as an element supporting volunteer behavior, understood as a voluntary and deliberate act of helping intended to benefit others, overcome difficulties, or meet needs. It is recognized for its multifaceted nature and its fundamental importance for society, the improvement of coexistence, and human progress (Pérez et al., 2021). Consistent with this, volunteer behavior can encompass a variety of activities, which may or may not have an altruistic component. However, its link to disaster risk management is highlighted, as it includes actions such as rescue, assistance, donations, social support, emotional containment, logistics, and collaboration (Martí-Vilar et al., 2019). Prosocial behavior has been predominantly studied in terms of types of assistance, with far less focus on the causes motivating this behavior. This indicates a weak association between volunteer activities themselves and their dispositional components, understanding that a volunteer action may have different values motivating factors for one person compared to another performing the same volunteer action (Padilla-Walker et al., 2022). This equation can develop differently according to diverse sociocultural contexts, the objectives of this behavior, and underlying motivations (Shadiqi et al., 2022).

Building on the above, disaster risk management within volunteerism can be viewed as a central issue, leading studies on volunteerism and prosocial behavior to suggest the possibility of investigating volunteers’ perceptions, values and emotions to understand prosocial behavior in both spontaneous and institutionalized volunteers (Marta et al., 2010).

However, it is essential to highlight that beyond individual psychological elements such as values and emotions that drive volunteers toward disaster response, the theory of social identity emerges as a crucial framework for understanding volunteer mobilization in disaster contexts (Tajfel & Turner, 1986/1989). This theory emphasizes the social identities of individuals, proposing that the self-concepts derived from the social categories to which one believes they belong shape their identity (Drury, 2012; Alvarado-Ardiles et al., 2019). In disaster risk management contexts, particularly during and after disasters, both affected groups and volunteer groups mobilize based on identity-related psychological constructs rooted in group belonging. These constructs can explain social support and, consequently, coping mechanisms, responses, and overall disaster risk management (Tajfel & Turner, 1986/1989; Paton, 2019).

Social representation is a form of knowledge that is socially constructed and shared, with a practical aim that contributes to the construction of a common reality for a social group (Wagner, 2015). Moscovici (1979) conceptualizes social representations as an organized system of knowledge that facilitates communication and integration within social groups, enabling the understanding and transformation of physical and social reality.

From a structural perspective, it is conceived as both a product and a process of mental activity through which an individual or group reconstructs the reality they face, assigning it specific meaning and structure regarding a particular phenomenon (Abric, 2001).

It is important to understand risk management in a way that allows it to be seen as a social representation with a defined structure emerging from the complex interaction between knowledge, beliefs, and attitudes of first responder volunteers, thereby enabling better performance in this task by volunteers. Social representation studies and their structural approach provide an epistemological and theoretical framework that allows the configuration of a network of connections between socio-natural disaster risk management and first emergency responders (Lo Mónaco et al., 2017).

Finally, as the current environmental context in Latin America and Chile has become conducive to the occurrence of various socio-natural disasters, it is essential to delve into the understanding of human psychosocial variables involved in disaster risk management. The objective of this study is to explore both the prototypical and categorical structure of the social representations of formal and spontaneous volunteers in socio-natural disaster risk management contexts, focusing on both similarities and differences.

## 2. Materials and Methods

The focus of this study is grounded in social representation theory, which allows for an understanding of human behavior through social norms, beliefs, and specific contexts (Jodelet, 1986). From this perspective, social representation theory is suitable for achieving a comprehensive understanding of behavior by including the role of beliefs, mental states, and social norms that influence human actions. Social representations manifest in actions, beliefs, and discourse, reflecting the dynamic nature of human groups and the specific phenomena that affect them (Moscovici, 1979).

The type of study adopted is exploratory and is based on the central core theory of social representations, which distinguishes between a stable core, consisting of consensual elements that determine the meaning and stability of the representation, and peripheral elements, which organize and complement the core, reflecting the group’s structure of values and norm (Abreu & Gaskell, 2015). This approach involves a flexible design that combines qualitative and quantitative techniques for data collection and analysis, aligning with the exploratory nature of the study (Moliner & Abric, 2015). This approach is in line with Dénzin and Lincoln (2012), who argue that in exploratory research, combining quantitative and qualitative methods allows for addressing complex questions and providing a deeper analysis of the phenomena studied through adherence to a socio-critical paradigm.

The study sample was obtained through non-probabilistic convenience and chain sampling, gathering information from a total of 101 participants, divided into 50 spontaneous volunteers and 51 formal volunteers, mainly volunteer firefighters residing in central-southern Chile, as shown in Table 1. Specific inclusion criteria were applied for each group: formal volunteers had to be affiliated with a disaster response institution, such as Non-Governmental Organizations or fire departments, while spontaneous volunteers had to be unaffiliated as volunteers with any institution and had to have participated in socio-natural disasters within the past three years.

**Figure 1:**
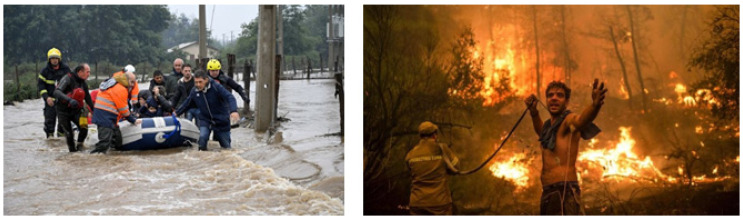
Inducing images (stimulation) for social representations survey. Note: Image one from left to right, disaster response by formal and spontaneous volunteers in Chillán and. Provided by Illustrious Municipality of Chillán. Image two from left to right, disaster response by formal and spontaneous volunteers in Greece. Excerpted from El Clarín Website (El Clarín, 2021).

Data collection was conducted through a self-administered digital questionnaire divided into three main areas: sociodemographic analysis, free word evocations, and hierarchical word association. The free evocations involved asking participants to respond with words, concepts, ideas, or emotions evoked by the presented stimulus, which in this case corresponds to a visual stimulus represented by Figure 1.

Data processing initially involved using an Excel spreadsheet with a list of the five words evoked by each participant in one column (A), followed by the participant number who evoked each word respectively in the next column (B), and in column (C) the number representing the order of evocation from 1 to 5, where 1 represents the most spontaneously evoked word and 5 the last evoked element. Following this, the number of distinct words, total word frequency, and total frequency of core words were extracted using the “count” function.

Words with a frequency of less than two were excluded from the data analysis due to their distance from the core and the potential distracting effect they might exert. A lexicographic analysis of the evoked words was performed, grouping conceptually and semantically similar or closely related terms, despite morphological differences (Rodríguez-De Ávila, 2019). The lexicographic analysis was conducted by verifying meanings using the online Royal Spanish Academy Dictionary, recognizing the semantic nature of the exercise to capture the maximum informative content from the evocations (Piermattéo et al., 2018).

After completing the lexicographic analysis and obtaining word frequencies, the data were prepared for transfer to SPSS 24 software. Descriptive and mean comparison analyses were conducted to calculate the mean evocation ranks of each distinct word and the accumulated frequency. The software was also used to analyze sociodemographic data.

The prototypical analysis of the representation was conducted by organizing the words based on the intersection of evocation rank and word frequency in a four-quadrant chart, including a core and three peripheral zones. Words with higher frequency and lower evocation rank were placed in the core, words with higher frequency and higher rank in the first periphery, and words with lower frequency and lower evocation rank in the second periphery (Abric, 2001). To identify the most relevant concepts for each volunteer group, frequency and evocation rank thresholds were determined to differentiate between core and peripheral words in the representation (Moliner & Abric, 2015). For frequency, a quadratic logic was applied, using the most frequent word divided by four to set the threshold. The evocation rank was determined as half of the maximum possible number of evoked words. This approach allowed the identification of both consensual elements and those representing more specific individual perspectives based on their position within the representational structure (Flick et al., 2015).

On the other hand, from the categorical analysis perspective, an inductive process was conducted through data-driven coding, guided by an audited procedure (Gibbs, 2013). Grounded theory aspects were utilized to enable the formation of analytical categories that group concepts based on their theoretical relationship, facilitating the interpretation of social representations through these categories of analysis (Flick et al., 2015; Gibbs, 2013). To strengthen the description of the representations, a category weight analysis was performed. This involved identifying three relevant categories of the representation: the category with the highest number of distinct words, the category with the highest absolute word frequency, and the category containing the largest number of core words in the representation. This analysis reinforces the structural prototype of the representation (Carrascal, 2008).

Finally, after conducting the analyses for each technique, a data triangulation exercise is proposed to enhance the descriptive, comprehensive, and explanatory power of each technique, facilitating their subsequent integration for drawing conclusions based on both exercises (Mason, 2006).

## 3. Results

### 3.1. Results: Prototypical Analysis in Formal and Spontaneous Volunteers

**Figure 2:**
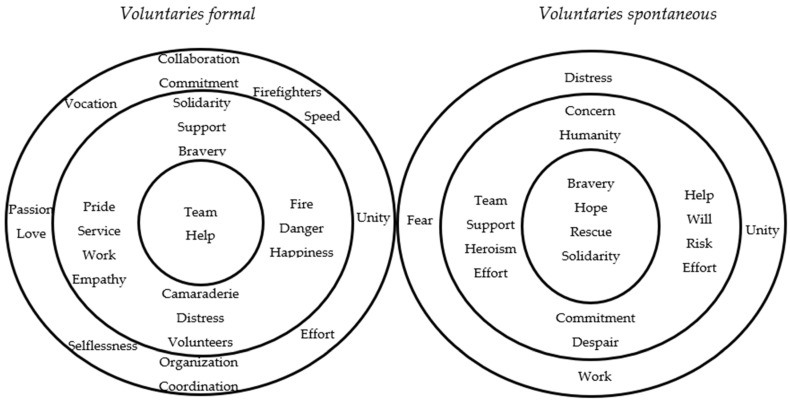
Comparative Prototypical Analysis in Formal and Spontaneous Volunteers: The prototypical analyses illustrated in the four-quadrant table are found in Annex 2. with frequencies of three or less were excluded from the figure in the third periphery. Note: Own elaboration.

The comparative prototypical analysis of social representations in formal and spontaneous volunteers examines how these two groups perceive and understand their roles and experiences in volunteering contexts differently. Additionally, it allows for the identification and understanding of the differences and similarities in the prototypical structures of social representations, reflected in the cognitive, motivational, value-oriented, and behavioral elements characteristic of each group. This analysis was carried out graphically through Figure 2, which represents the prototypes in concentric circular figures, with the central part understood as the core and the outer parts as their respective peripheries.

In the core representation of formal volunteers, the concepts of “Help” and “Team” are central. These form the core nucleus—a small, concentrated core that represents the most stable and fundamental part of this representation. These terms capture the essence of formal volunteerism, emphasizing its collaborative nature and primary goal of providing assistance in emergency situations. Emotional and value-oriented qualities that motivate formal volunteers in relation to aid and assistance, such as “Solidarity”, “Empathy”, “Pride”, “Support”, and “Courage”, are present. Additionally, emotional elements produced during the volunteer’s actual work are observed, reflecting both positive and negative aspects, such as “Happiness”, “Distress”, and “Pride”. Understanding how the first periphery shapes the core, the concept of “Team” is enriched by elements like “Comradeship”, which directly connect to the core through a highly cohesive process on a social and networked level. Other concepts such as “Service”, “Work”, and “Danger” are also observed, identifying and interpreting that formal volunteers perceive their role as a serious, structured service or work with a high level of commitment to the community, often performed in risky contexts.

In contrast, for spontaneous volunteers, the core elements are “Courage”, “Solidarity”, “Hope”, and “Rescue”. The first element underscores the importance of spontaneous volunteerism as a high-cost prosocial action, despite the potential danger involved, which can be characterized by a strong positive association. The second and third elements refer to values that act as driving forces behind actions toward desired outcomes, as well as the representation and emotion generated by volunteering and its connection with affected communities. Finally, the concept of “Rescue” refers to the actions and functions associated with the stimuli, encompassing the rescue activity performed by volunteers, which requires certain expertise or technical skills for optimal performance. In the first periphery, elements such as “Heroism”, “Will”, and “Humanity” shape the core for spontaneous volunteers, highlighting a commitment to others, particularly in the concept of courage, and strongly supporting the heroic and courageous role of the volunteer in disaster situations. This framework reveals a motivational inclination based on ego-related implications and the ideal of the volunteer as a recognized role model, reflecting that, in practice, they respond to human needs through sheer will.

### 3.2. Results: Categorical and Weight Analysis in Formal and Spontaneous Volunteers

Table 2 presents the analysis of the weight of categories compared between formal and spontaneous volunteers. A total of seven categories (Social Capital, positive Emotions/Feelings, negative Emotions/Feelings, values, volunteers’ perception, natural elements, and volunteer behavior) were created inductively using a data-driven coding technique, in which the evoked words were coded and assigned to the generated categories based on the collected information. Subsequently, following the criteria of the categorical analysis of social representations—such as word frequency, frequency of evocations, and frequency of evocations of core words—the categories with the highest presence according to each criterion were identified. This resulted in three prominent categories for formal volunteers and two for spontaneous volunteers.

The first criterion of word frequency reveals a tie in the weight of the categories “Volunteer Perceptions” and “Volunteer Behavior”. The first category pertains to how formal volunteers characterize their work and personal identity. In dialogue with the prototypical analysis of representations, a notable relevance emerges regarding self-perception of their role, primarily associated with the attitudes required for volunteering in risky contexts. This includes a symbolic set of behaviors and values grounded in ethical codes and the history and identity and attachment of the organization. The second category, based on the word frequency criterion, highlights the capacity for coordination and organization of resources and tasks as a central element in the behavior of formal volunteers. It also underscores proactivity and consideration for others, whether team members or third parties. This category is also reflected in the criterion of evocation frequency, emphasizing that the representation of formal volunteers is strongly constructed based on how they conceive their behavior.

On the other hand, the criterion of frequency of evocations of core words identifies “Social Capital” as a central component. This concept relates to the ability to engage in dialogue and articulation, as well as the capacity to view volunteer work as a social action. This not only involves a commitment to society but also a strong bond with colleagues and a constant concern for others. This aspect is reinforced in the prototypical results, where the logic of cohesive teamwork is highlighted, along with the importance of articulation and networking for effective disaster risk response, thereby framing volunteering as a collaborative effort.

Regarding the analysis of category weight for spontaneous volunteers, the category “Values” predominates across all three weight criteria, while “Volunteer Behavior” stands out in the word frequency criterion. This highlights the direct influence of ethical and moral principles, marked by a strong prosocial component. Spontaneous volunteers express their desire to help others or work for relevant causes based on their values. This value-driven orientation may stem from various types of values associated with spontaneous volunteering, generating a solid, cohesive, and coherent categorical representation.

Furthermore, although spontaneous volunteers lack technical and organizational knowledge in disaster response, they exhibit significant emphasis on actions and technical behaviors related to disaster management. In this case, behavior becomes a relevant aspect for this group, highlighting their commitment and willingness to act in emergencies.

### 3.3. Results: Comparative Analysis of Representations in Formal and Spontaneous Volunteers

The representation of formal and spontaneous volunteering reveals differences in terms of characterization, response, and relationship to disaster situations.

First, it is observed that in the representation of spontaneous volunteering, technical behavior in disaster response is mentioned more frequently, whereas, in formal volunteering, this perspective is less common. This suggests that spontaneous volunteers conceive disaster response in technical terms, even though they lack training and a clear understanding of the nature of their role. Formal volunteers, on the other hand, express their representation through networking, resource management, and teamwork, characterized by their ability to coordinate and collaborate effectively in managing socio-natural disaster risk. They operate within organized structures with clearly defined codes and roles.

In terms of characterization, formal volunteering is self-defined by emphasizing institutional codes and their role in fulfilling specific tasks rather than explicit values or direct emotions. While the importance of values is acknowledged, their focus remains on coordination and organized management. In contrast, spontaneous volunteers exhibit a pronounced inclination toward prosocial values and an idealized vision of the volunteer as a hero, distinguishing themselves by their orientation toward moral and prosocial principles, which drive their actions to intervene in disaster situations.

Finally, spontaneous volunteers often respond in an isolated and impulsive manner until they can integrate into an organizational process, which limits their impact compared to their performance when part of organized groups. Their effectiveness largely depends on their ability to integrate into teams and collaborative networks. Formal volunteers, in turn, make cohesion and coordination the driving forces behind disaster risk management, minimizing the presence of technical elements in their representation of response and prioritizing collaborative work.

## 4. Discussion

As an initial conclusion, before proceeding with a thematic discussion, it can be noted that the increasing physical and psychosocial impacts of socio-natural disasters linked to climate change have prompted various behaviors among citizens who volunteer during emergencies. These actions are driven by social identity, values, and the emotional transfer from affected human and non-human victims, as well as institutional norms and human coordination. This scenario has fostered an empathetic call for assistance on behalf of those affected, marked by a strong prosocial and value-driven response from volunteers in disaster contexts where the demand for help is high despite the risks involved.

### 4.1. Adherence, Permanence and Forms of Volunteering

Based on the data and literature, it is suggested that we are in a phase of challenges and opportunities related to volunteering in the context of socio-natural disaster risk management, particularly within a scenario marked by contemporary social changes, such as increased individualism, urbanization, migration from rural areas, and population aging (Espinosa et al., 2011). Although a decline in international volunteer rates has been observed, this phenomenon is argued to represent a transformation rather than a decline, where motivations and ways of volunteering are being redefined (Whittaker et al., 2015). Therefore, the limits of adherence to volunteerism call for volunteer institutions to rethink volunteering and its connection to individuals’ motivational factors, which can change over time and differ between formal and spontaneous volunteers.

Similarly, formal volunteerism is characterized by a consistent and demanding commitment to a specific organization, based on altruistic values and a strong institutional commitment (Arianti & Koentjoro, 2023). However, recruiting and retaining these volunteers remains a significant challenge for disaster response organizations such as fire departments and disasters organizations. These institutions need to reconsider their approach to volunteering and adapt to current societal demands, not only in emergency response but also in terms of adherence to this specific form of volunteerism, especially in disaster contexts. Therefore, based on the findings of this study, it is suggested that adherence be evaluated through psychological selection criteria alongside institutional criteria to ensure suitability for disaster risk management tasks. This evaluation should assess individual and collective capacities, motivations, and suitability to better match volunteer roles with the specific Chilean risk context, where responses are both operational and administrative (Grant & Langer, 2021).

For spontaneous volunteerism, it is recommended to implement simulation exercises and pre-disaster activities to familiarize these volunteers with decision-making processes and the logistical management of volunteer resources (McLennan et al., 2016). This will not only contribute to better preparedness but also optimize participation in socio-natural disaster situations. Given the prominence of social representation through the values, emotions, and attitudes of spontaneous volunteers, it is essential to develop collective action plans based on the knowledge and resources available within affected communities, fostering coordination and a sense of belonging prior to disasters, thus creating disaster-prepared communities (Bier et al., 2023).

Integrating formal and spontaneous volunteers remains a challenge due to the lack of effective coordination that considers volunteers outside organized systems. Recognizing the active agency of citizens is crucial for planning targeted actions that optimize participation from both spontaneous and formal volunteers, aligning expectations and promoting greater cohesion and longevity in service. However, this agency should be organized to distribute specific roles suitable for each volunteer (United Nations Office for Disaster Risk Reduction [UNDRR], 2015; Whittaker et al., 2020). This implies not only the effective inclusion of volunteers in risk management plans but also the ability to integrate new forms of volunteerism that can shape potential spontaneous volunteer figures who do not currently have a permanent affiliative space (Wilson, 2012). Understanding how these new forms of volunteerism can be effectively managed will help maximize their impact and sustainability in emergencies (Barsky et al., 2007).

This approach helps counter the impulsive and generally uncoordinated response of spontaneous volunteer action through the establishment of connections, follow-up, and periodic informational dialogues by formal groups or institutional actors with potential volunteers. The goal is to establish non-affiliative communication spaces through sporadic interaction without commitment, with communities susceptible to socio-natural disaster risk. This can create a registry for and slight coordination within this potential volunteer force, while also providing basic tools for an adequate response, allowing them to perform community action more effectively in disaster contexts, taking into account their particular characteristics (Marta et al., 2010; Fernández et al., 2006).

### 4.2. Values, Emotions and Prosocial Behavior in Formal Voluntary Responders and Spontaneous Voluntary Responders

While values are a key category and element within the representation of formal and spontaneous volunteers, it is pertinent to discuss the distinctions that may arise among different types of values. Freise and Walter (2024) propose a classification that includes religious values, socially transformative values, reciprocity values self-efficacy, and community concern values collectivism.

In line with this, the findings of this study align with the literature, indicating that collectivism and self-efficacy values are significantly associated with prosocial behavior in socio-natural disaster contexts. These values play a significant role in shaping prosocial behavior, whereas values associated with individualism and selfish motives show no significant relationship (Shadiqi et al., 2022). However, particularly in the case of spontaneous volunteering, an approach to volunteer work can be observed that arises not necessarily from prosocial elements but rather from the mobilization triggered by negative environmental contexts affecting society.

Emotions also play a crucial role in the values, motivation, and performance of volunteers. For formal volunteers, preparing for and executing tasks in disaster risk contexts generate positive emotions, such as joy, which influence their satisfaction and motivation. However, this raises a concern, as these positive emotions may stem more from the inherent risk of the tasks than from the act of volunteering itself. Thus, it is important to analyze the origins of these emotions and their motivational implications (Arias, 2015).

On the other hand, based on representations, it is proposed that spontaneous volunteers face significant emotional burdens and challenges in their disaster response. These challenges are linked to their perceptions of the disaster, lack of formal training, absence of prior experience, and the exercise of empathy associated with prosocial behavior (Gowan et al., 2014). However, as highlighted in a study on volunteer assignment factors conducted by Xue et al. (2024), optimizing volunteer selection during disasters involves considering elements such as the volunteers’ willingness to respond, their skills and competencies, the specific needs of the affected site concerning the task, and the time satisfaction of disaster victims. Notably, the study underscores the absence of emotional factors as a significant consideration in spontaneous volunteerism, recognizing its relevance in disaster response.

In this context, it is suggested that citizens are motivated to volunteer during disasters not solely out of altruism but also as a means of regaining emotional balance lost during the event, improving their subjective well-being, and fulfilling the psychological demands of risk experiences.

This finding aligns with previous research and the work of Innes et al. (2024), who emphasize the significance of values and emotions, both in their expression and regulation, within disaster contexts for volunteer groups. These elements are not merely behavioral guides but also serve as distinct motivational factors for engagement, participation, and continuity in both formal and spontaneous volunteerism in disaster risk management contexts. However, it is suggested that prosociality, internal values, and emotions represent only a portion of the psychological dimension of volunteering in disaster settings.

In discussing social identity theory in disaster contexts through social categories, different forms of volunteering are established, viewed here as a social group. According to Benedict (2022), in a study on spontaneous volunteering during wildfires in California, volunteer groups without prior coordination negotiated their personal identities and formed categories that unified them based on specific tasks for organizing emergency response, resilience, and/or recovery efforts. In this case, the social representation of spontaneous volunteers highlights a limited emphasis on cohesion, identification, and coordinated work among volunteer groups. This raises concerns about the risks of disconnection and individual efforts, where behavior is not negotiated but rather driven by internal, intuitive, and spontaneous psychological dimensions such as values and emotions.

However, it is proposed that, considering social identity theory has primarily been addressed from experimental perspectives often overlooking environmental elements (Valera & Pol, 1994), the context of socio-natural disasters shaped by issues like climate change presents differentiated impacts and responses depending on territorial relationship and settings, such as rural versus urban areas (Peña-Garay & Sandoval Díaz, 2024). From the perspective of environmental psychology, it has been suggested that these territorial contexts, marked by elements like urban social identity, can generate identity categories linked to space (Valera, 1997). These categories, in turn, encourage mobilization and motivate individuals who do not share a collective social identity to provide assistance in disaster contexts. This behavior is triggered by the desire to protect a territory associated with their identity (Paton, 2019; Vidal et al., 2013).

### 4.3. Social Capital, Teamwork and Formal Volunteer Responders

From the perspective of formal volunteers, it is suggested that individuals’ identification with a specific goal can significantly influence their behavior (Perrier et al., 2024). Social identity theory aligns closely with this group, given the institutional relationships inherent in formal volunteering spaces. These environments provide an identity framework based on constructed categories, fostering social identification that reflects how individuals integrate collective attributes into their self-concept.

Institutions play a central role in this process by shaping a cohesive identity among their members through customs, codes, partnership, symbols, responsibilities, obligations, and material signs of affiliation (Nowakowska, 2023). This identity contributes to the construction of role clarity and definition. In the context of this study, the role of volunteer firefighters exemplifies this dynamic, as cognitive mobilization enables effective, coordinated, dialogued, and cohesive responses to disasters. Furthermore, the importance of social capital within this group is highlighted as a key element in disaster risk management (Paton, 2019; Perrier et al., 2024).

Social capital, understood as social cohesion, the bonding among individuals, and the formation of social networks, is configured as a crucial element in the management of socio-natural disaster risks, particularly by formal volunteers. This concept underpins voluntary behavior, as it facilitates cooperative and organized responses to emergency situations (Cárdenas-Briones, 2020). The study results show that formal volunteers particularly value social capital when affiliated with organizations that promote relationship networks and a sense of belonging. This strengthens human bonds within volunteerism, enhancing communication and coordination during emergencies.

Formal volunteers, with their organized and systematic approach, demonstrate greater effectiveness and safety in their interventions, leveraging their social capital through cohesion and adequate preparation that facilitates collaboration and resource management (Bachner et al., 2016). This orientation, linked to social capital, raises awareness about its development among spontaneous volunteers, suggesting an intermediate organizational structure between formal and spontaneous volunteering. Such a structure would highlight the actions, impact, and importance of social cohesion in behavior during socio-natural disasters.

Aligned with the perspective presented by Bachner et al. (2016), teamwork and the development of social capital are reflected in actions such as helping, sharing, cooperating, and organizing, which foster the creation of strong social bonds both before and after a disaster, enabling these connections to remain functional across all phases of risk management. From this standpoint, social capital emerges as a critical factor for disaster mitigation, response, and adaptation, supporting an integrated risk management approach for responders. However, the strong social ties characteristic of responders with less formal organization, identity, and structure may lead to overlooking risks associated with disaster response, ultimately hindering effective disaster risk management (Rodríguez-Garzón et al., 2021).

However, the adoption of social capital as a relevant element within the social representation of formal volunteerism stems from the emphasis placed on establishing positive peer relationships and ensuring appropriate organization and collaboration among them for an effective response to socio-natural disaster risks. Knowledge and skills are subordinated to the demand for social capital from communities and formal responders, positioning teamwork in volunteer efforts as essential, above the technical execution of tasks or preparedness. Articulation and communication are considered more important than preparedness and training, although teamwork must be carried out in parallel with proper training.

### 4.4. Heroic Characterization of the Spontaneous Volunteer Responder

The study results indicate that spontaneous volunteering in socio-natural disaster risk contexts tends to be shaped by a heroic perception of its role. This predominant representation highlights the bravery and fearlessness of volunteers, as well as their heroism (Daddoust et al., 2021).

Conceiving this heroic ideal of volunteering may overlook personal interests and lead to saturation in aid spaces, reinforcing the image associated with volunteering. However, this vision tends to obscure the potential negative consequences of such idealization, including emotional impacts and erratic behaviors that may arise during crises (Haynes et al., 2020).

Spontaneity, conceived as heroic and courageous, may result in a lack of care, continuity, and adherence to safety protocols, thereby increasing risks for this group. In light of this, it is essential to critically integrate new ways of conceptualizing spontaneous volunteering in changing environmental, social, and institutional contexts. With increasingly diverse instances of spontaneous volunteering, it is possible to articulate and pre-organize these efforts to enhance coordination of the potential spontaneous volunteer workforce (Marta et al., 2010; Haynes et al., 2020).

It is crucial to approach the technical idealization of volunteering in disaster contexts with caution, especially regarding spontaneous volunteering. In these contexts, adopting certain flexible strategies used by formal volunteering frameworks is proposed, focusing on communication, dialogue, training, and a clear definition of roles, rather than viewing volunteering in disaster risk contexts as merely the execution of specialized and emergent technical actions (Paciarotti & Cesaroni, 2020; Jaime et al., 2023).

Finally, the heroic characterization of volunteers, while potentially serving as a source of motivation and aspiration for individuals, may also pose underestimated risks, particularly for spontaneous volunteers. Assuming this role can lead to an underestimation of the inherent risks of disaster situations, endangering both their safety and the effectiveness of emergency responses.

### 4.5. Limitations and Projections

In methodological terms, the prototypical and categorical analysis of social representations presents challenges, particularly in establishing a theoretical consensus on thresholds for rank and frequency, which may affect the reliability of conclusions (Lo Mónaco et al., 2017; Dany et al., 2015). Additionally, the lack of access to tools such as the EVOC3000 program in Latin America limits the use of automated methodologies, necessitating the manual application of analytical techniques, which increases the workload for researchers (Carrascal, 2008; Vergès, 1992). From a methodological perspective, it is proposed to strengthen the representational structure through the comparison of means of the evoked and ranked words within the representational groups. This would allow for a more precise definition of representational structures in studies of social representations (Dany et al., 2015).

From a theoretical perspective, there is confusion between the terms “spontaneous volunteer” and “unaffiliated volunteer”, highlighting the need for more precise classifications in the literature. Additionally, there is a lack of characterization of disaster or emergency responders, particularly regarding volunteering and disaster risk management (Martínez et al., 2021).

Furthermore, analyzing the performance of spontaneous volunteers in the context of disaster risk management from the perspective of social identity based on territory and environment could uncover elements previously overlooked in this research (Valera & Pol, 1994). These findings would be valuable for broadening the understanding of spontaneous volunteer mobilization beyond individual psychological particularities (Benedict, 2022).

As a forward-looking proposal, it is suggested to reconsider the motivational axis of volunteering to prevent idealization from interfering with institutional duties during emergencies. It is also essential to formalize and educate about the role of volunteers in disaster situations to avoid disaster tourism (Mendes & Sonaglio, 2013).

Additionally, from a disaster risk governance perspective, where the state plays a key and responsible role, prevention and education must be seen as essential factors in the development of organizational capacities for voluntary actions (Shi, 2012). This approach promotes formative learning about emergency management, enabling the identification of motivations, fostering affiliation and longevity in volunteer service, and encouraging attitudinal changes (Omoto & Snyder, 1995). Such a framework enhances reflective capacities and contributes to effective organization for disaster risk management, involving both state and public actors in a collaborative effort.

Projections for future studies suggest four areas of action. First, longitudinal studies are recommended to explore the representations of individuals transitioning from spontaneous to formal volunteering (Kankanamge et al., 2019). Second, it is important to delve into the psychosocial dimensions of volunteering, such as motivation and adherence, and to explore new forms of volunteering to identify emerging modalities of participation (Kragt & Holtrop, 2019). Third, investigating the influence of gender on adherence to volunteering in disaster risk contexts is suggested, focusing on identifying differences or similarities in motivations and participation, as well as how gender impacts group dynamics, roles, and leadership (Gurung et al., 2019). Fourth, it is necessary to analyze the incidence of multiple and family volunteering, as this type of participation could reveal psychosocial and cultural characteristics, particularly in Chile and Latin America, that have been underexplored and are relevant for understanding the phenomenon of volunteering from a sociocultural perspective linked to family and territory (Kamerāde, 2023).

## Figures and Tables

**Table 1:** Socio-demographic characterization of the sample.

Characteristics	Formal Volunteers 50.5% (51)	Spontaneous Volunteers 49.5% (50)	Total 100% (101)
Gender			
Female	33.3% (17)	54% (27)	43.6% (44)
Male	64.7% (33)	44% (22)	54.5% (55)
No-Binary	2% (1)	2% (1)	2% (2)
Ocupation			
No-ocupation	5.9% (3)	0	3% (3)
Student	35.3% (18)	72% (36)	53.5% (54)
Dependent worker	47.1% (24)	12% (6)	29.7% (30)
Self-employed	11.8% (6)	16% (8)	13.9% (14)
Territory			
Rural	19.6% (10)	32% (16)	25.7% (26)
Urban	80.4% (41)	68% (34)	74.3% (75)

Note: Own elaboration.

**Table 2:** Analysis of the Weight of Categories for Formal/Spontaneous Volunteers.

Criteria for Category Weight	Salient (Relevant) Categories of Formal Volunteers		Salient (Relevant) Categories of Spontaneous Volunteers	
Word Frequencies	Perceptions of the Volunteer	Volunteer Behavior	Values	Volunteer Behavior
Frequency of Evocations	Volunteer Behavior		Values	
Frequency of Evocation of Core Words	Social Capital	Volunteer Behavior	Values	

In the criteria for word frequency and the frequency of evocations of core words, there was a tie, leading to the identification of two salient categories in some cases. Note: Own elaboration.

## Data Availability

The data statement information can be downloaded at: https://drive.google.com/drive/folders/1AyFXLK8H-6CH18AuDBNARG6_0SkNfVvN?usp=sharing.
